# Boredom belief moderates the mental health impact of boredom among young people: Correlational and multi‐wave longitudinal evidence gathered during the COVID‐19 pandemic

**DOI:** 10.1111/jopy.12764

**Published:** 2022-08-21

**Authors:** Katy Y. Y. Tam, Christian S. Chan, Wijnand A. P. van Tilburg, Iris Lavi, Jennifer Y. F. Lau

**Affiliations:** ^1^ Department of Psychology The University of Hong Kong Hong Kong China; ^2^ Department of Psychology King's College London London UK; ^3^ Department of Psychology University of Essex Colchester UK; ^4^ Department of Psychology University of Bath Bath UK; ^5^ School of Social Work University of Haifa Haifa Israel; ^6^ Youth Resilience Unit, Wolfson Institute of Population Health Queen Mary University of London London UK

**Keywords:** adolescents, boredom, COVID‐19, emotion beliefs, mental well‐being, young adults

## Abstract

**Objectives:**

Young people's experience of boredom and its psychological health sequelae have been exacerbated by the COVID‐19 pandemic. The present study examined the moderating role of boredom beliefs—the extent to which one affectively dislikes boredom (*boredom dislike*) and cognitively accepts it (*boredom normalcy*)—on the association between boredom experience and mental well‐being. We also validated a new measure of boredom beliefs in two different samples of young people.

**Method:**

We report data from a correlational study with British young people aged 12–25 (Study 1; *N* = 2495) and a 16‐week eight‐wave within‐subject study with Israeli adolescents aged 12–18 (Study 2; *N* = 314).

**Results:**

Across both studies, disliking boredom was associated with higher frequency and intensity of boredom. Boredom dislike moderated the negative association between boredom and mental well‐being, such that the association was more salient among those who strongly disliked boredom. Normalizing boredom was positively associated with mental well‐being. The measure of boredom beliefs demonstrated fair validity and reliability.

**Conclusion:**

Results provide novel insights into the potential buffering effect of boredom beliefs against the mental health impact of boredom, particularly at a time of reduced activity. These findings generalize across two different countries.

## 1 INTRODUCTION

Protracted boredom can lead to undesirable outcomes in young people, including lower life satisfaction (Spruyt et al., 2018), depressive feelings (Spaeth et al., 2015), youth deviance (Malizia, 2018), and risk behaviors such as binge drinking and internet addiction (Biolcati et al., 2018). Because young people are more prone to boredom (Caldwell et al., 1999; Weybright et al., 2020), they might be especially challenged by the constraints on autonomy and leisure in the coronavirus disease 2019 (COVID‐19) pandemic. Identifying those who are particularly at risk may help mitigate the adverse impact of boredom on well‐being and enhance preparedness for similar high‐risk situations in the future. There is emerging research suggesting that the deleterious effect of negative emotions on health is more pronounced among people who do not see value in these emotions (Ford & Gross, 2018; Luong et al., 2016). Given that people vary in their lay beliefs about boredom (Tam, Van Tilburg, & Chan, 2021a), are young people more susceptible to the mental health impact of boredom if they hold this common emotion in low regard? The current research addressed this question.

## 2 BOREDOM AND MENTAL WELL‐BEING

Boredom is an emotion that is typically experienced in monotonous (Daschmann et al., 2011), unengaging (Hunter & Eastwood, 2016; Yakobi et al., 2021), unchallenging (M. B. Harris, 2000; Van Tilburg & Igou, 2012), meaningless (Chan et al., 2018; Van Tilburg & Igou, 2012) circumstances with a perceived lack of autonomy (Van Hooft & Van Hooff, 2018; for review, see Tam, Van Tilburg, Chan, Igou, & Lau, 2021). It can be defined as an aversive state of wanting to, but being unable to, engage in a satisfying activity (Eastwood et al., 2012). People's cognitive abilities in attentional engagement, perceived constraints, and abilities to identify satisfying activities are central to the experience of boredom. These components might be the reasons why young people are argued to be especially prone to boredom (Caldwell et al., 1999; Weybright et al., 2020), given that they are undergoing maturational changes in relevant cognitive (Luna et al., 2004) and emotional abilities (Yurgelun‐Todd, 2007), with an increased desire for autonomy (Daddis, 2011) but inadequate skills to structure their free time (Caldwell et al., 1999), to exert self‐control (Casey & Caudle, 2013), and to cope with boredom (Spaeth et al., 2015; Weybright et al., 2020). Indeed, boredom is a common experience among youth (Chin et al., 2017; Larson & Richards, 1991; Spaeth et al., 2015).

While boredom, like other emotions, comes and goes, chronically experiencing it can potentially be detrimental to one's well‐being. Boredom proneness is a disposition that reflects the frequency and intensity of boredom experiences, and one's perception of how boring their life is (Tam, Van Tilburg, & Chan, 2021b). Boredom proneness is associated with symptoms of depression, anxiety (e.g., Fahlman et al., 2009; Goldberg et al., 2011), stress (Lee & Zelman, 2019), somatization, interpersonal sensitivity, obsessive–compulsive tendency (Sommers & Vodanovich, 2000), apathy, anhedonia (Goldberg et al., 2011), and lower life satisfaction (Tam, Van Tilburg, & Chan, 2021b). Also, it is related to a wide range of risk behaviors, including risky driving (Oxtoby et al., 2019), emotional eating (Crockett et al., 2015; Mercer‐Lynn et al., 2013), and problematic smartphone use (e.g., Elhai et al., 2018; Ksinan et al., 2019).

For young people in particular, the evidence on the diminished self‐control at this developmental stage (Casey & Caudle, 2013), coupled with an interlocking relationship between boredom and self‐control (e.g., Bieleke et al., 2021; Tam, Van Tilburg, Chan, Igou, & Lau, 2021; Wolff et al., 2020; Wolff & Martarelli, 2020), underscore potentially heightened risk among them to respond to boredom with impulsive, risky behaviors, which might, in turn, be detrimental to their well‐being. Indeed, boredom proneness is found to be associated with deviant (Malizia, 2018) and problematic behaviors (Biolcati et al., 2018), lower life satisfaction (Spruyt et al., 2018), and depressive feelings (Spaeth et al., 2015) among young people.

Considering the developmental changes across adolescence and young adulthood, boredom may be particularly challenging for them under the constraints on autonomy and leisure activities during the pandemic. Studies on boredom and COVID‐19, thus far, predominantly focus on adult populations. Generally, it was found that people living in lockdown reported greater levels of boredom (Droit‐Volet et al., 2020) and that boredom was associated with various negative psychological outcomes (e.g., Boylan et al., 2021; Caci et al., 2020; Chao et al., 2020; Wolff et al., 2020). There is a paucity of data on boredom in young people during the COVID‐19 pandemic; the only study that has examined youth boredom focuses on the educational context (Martarelli et al., 2021). It is important to investigate factors that might help mitigate the mental health impact of boredom for young people, especially during high‐risk situations such as the pandemic.

## 3 BOREDOM BELIEFS

Emotion beliefs are the ways people think about emotions. There are two core dimensions of beliefs—whether emotions are good (or bad) and whether they are controllable (or not) (Becerra et al., 2020; Ford & Gross, 2018; Hong & Kangas, 2021). While there is a wealth of research on beliefs about emotion controllability (e.g., Ford, Lwi, et al., 2018; Kneeland et al., 2020; Tamir et al., 2007), less research has examined beliefs about the goodness of emotions (see a review by Hong & Kangas, 2021). Beliefs about the goodness of emotion refer to beliefs about whether an emotion is desirable, useful, helpful, or valued (Ford & Gross, 2019). For example, people tend to believe that positive emotions are more useful for self‐control than negative emotions (Tornquist & Miles, 2019); people tend to believe that anger, an unpleasant emotion, can be instrumental in confrontations (Sinaceur & Tiedens, 2006; Tamir & Ford, 2012).

Emerging research has underscored the importance of these emotion beliefs on emotional experience and well‐being (e.g., Ford & Gross, 2018, 2019; Ford, Lam, et al., 2018; Luong et al., 2016; Yoon et al., 2018). It is theorized that people who believe a particular emotion is bad more readily notice the signs of that emotion and perceive it as unpleasant, which in turn alters their emotional experience (Ford & Gross, 2018). Emotion beliefs may attenuate the emotion‐health link through altering emotional experience, the accompanying distress, and each stage of emotional regulation, such as identification of the need for regulation and selection of regulation strategies (Ford & Gross, 2019; Luong et al., 2016). These theoretical propositions are corroborated by empirical findings. For example, “liking” withdrawal emotions, such as fear and disgust, was found to be associated with less intense experience of these emotions (Harmon‐Jones et al., 2011). Valuing negative affects reduces their detrimental impact on health (Luong et al., 2016). On the contrary, negative attitudes toward emotion have a medium‐to‐large relation with higher depressive symptoms (Yoon et al., 2018).

People have distinct beliefs about different emotions (Ford & Gross, 2018; Harmon‐Jones et al., 2011). Based on the above findings, *lay beliefs about boredom* might similarly influence boredom experience and its deleterious effect on mental well‐being. The associations of rational and irrational evaluative beliefs with boredom proneness provide some insights (Milea et al., 2021). Boredom is a functional emotion that informs people of the current situation and motivates them in pursuit of something more beneficial (Bench & Lench, 2019), meaningful (Van Tilburg & Igou, 2012, 2017), engaging (Danckert et al., 2018; Eastwood & Gorelik, 2019; Tam, Van Tilburg, Chan, Igou, & Lau, 2021), and/or fulfilling (Elpidorou, 2014). Therefore, despite boredom being an unpleasant experience (Smith & Ellsworth, 1985; Van Tilburg & Igou, 2017), people can believe it to be a valuable emotion.

Indeed, preliminary data (Tam, Van Tilburg, & Chan, 2021a) shows that people vary in their boredom beliefs. Three key lay beliefs about boredom were proposed—*boredom functionality*, *boredom dislike*, and *boredom normalcy*. Boredom functionality is a behavioral component concerning the extent to which people recognize the functions of boredom. Boredom dislike is an affective component concerning the extent to which people affectively dislike boredom, which is akin to the constructs of affect valuation (Luong et al., 2016) and affective attitude toward emotions (e.g., how much do I like this emotion?; Harmon‐Jones et al., 2011). Boredom normalcy concerns whether people normalize the experience of boredom; normalizing emotions such as grief is a common regulation strategy applied in therapy (Dominick et al., 2010; Harris, 2010).

These beliefs concern how people *evaluate* boredom rather than how they *experience* or *respond to* boredom. They are distinct from the boredom experience itself, boredom coping (Hamilton et al., 1984; Nett et al., 2010, 2011), or boredom tolerance (i.e., people's response to the onset of boredom; Galla et al., 2020). For example, the belief that boredom is undesirable is different from tolerating or avoiding boredom; believing boredom is a normal emotion is different from engaging in cognitive reappraisals. There is a clear theoretical distinction between emotion belief and emotion regulation (e.g., Ford & Gross, 2018, 2019). Tam, Van Tilburg, and Chan (2021a) suggest that people who dislike boredom have a higher tendency to experience it. Yet, without a means to assess them, there has been relatively scarce research on boredom beliefs in young people.

## 4 CURRENT RESEARCH

The current research sought to investigate individual differences in young people's boredom beliefs, boredom experience, and mental well‐being using correlational (Study 1) and multi‐wave repeated‐measure (Study 2) data. Study 1 served as an initial test of the variables with a large sample of young people aged 12–25 in the UK. Additionally, we aimed to validate a new measure of boredom beliefs. Study 2 was an eight‐wave within‐subject study that examined these associations collapsed across 16 weeks in a sample of adolescents aged 12–18 in Israel. We targeted two facets of boredom beliefs, boredom dislike and boredom normalcy, and examined boredom experience in terms of its frequency and intensity (Tam, Van Tilburg, & Chan, 2021b). Because much research on boredom in the pandemic focuses on psychological distress (e.g., depression, anxiety; Chao et al., 2020), we examined this issue from a different angle through investigating the positive aspect of mental health: well‐being. Across the two studies, we tested two hypotheses: (1) disliking boredom is positively associated with frequency (H1a) and intensity (H1b) of boredom; and (2) the association of boredom frequency (H2a) and intensity (H2b) with mental well‐being is stronger among those who reported higher levels of boredom dislike. We did not formulate a hypothesis regarding boredom normalcy as there is limited research on the effect of normalizing emotions. The analyses for boredom normalcy were thus exploratory.

## 5 STUDY 1

The purpose of Study 1 was two‐fold. First, we investigated the association between boredom beliefs and boredom experience, as well as the role of boredom beliefs in attenuating the link between boredom experience and mental well‐being. Second, given that the Boredom Beliefs Scale (Tam, Van Tilburg, & Chan, 2021a) has not been administered in adolescent sample, we examined its psychometrics properties to ensure that it is a valid and reliable measure for hypothesis testing.

### 5.1 Method

#### 5.1.1 Participants and procedure

Data from this study were derived from a larger research project on emotional impact of the global COVID‐19 pandemic among adolescents and young adults. The study was approved by the Psychiatry, Nursing and Midwifery Research Ethics Committee at Kings College London (ref: HR‐19/20–18,868). Anyone aged between 12 and 25 residing in the UK at the time of data collection (from 12th May to 2nd December 2020) was eligible to take part. Participants were recruited via several methods: advertising within UK schools, colleges, and universities, research advertisement websites, social media, and charities. All participants aged 16 or over provided informed consent. For participants under 16, informed assent/consent was provided by participants and their parent/guardian, respectively. Participants were offered vouchers for their time spent taking part in this and subsequent follow‐up surveys. A total of 4872 respondents clicked on the survey link. Excluding those who (1) did not report anything other than initial demographic information (*n* = 1932), (2) were duplicate responses (*n* = 33), (3) did not meet age criteria (*n* = 13), (4) completed the survey in less than 5 minutes (*n* = 41; median completion time was 18 minutes), (5) were not in the UK (*n* = 48), (6) showed other evidence of inauthentic responding, such as irrelevant responses to qualitative questions (*n* = 245, identified by 3 independent coders), or (7) had missing data on key variables for this analysis (*n* = 65),^1^ the final sample contained 2495 young people (70.2% female^2^; age range = [12, 25], *M* = 17.9, *SD* = 3.58).^3^

#### 5.1.2 Measures

We administered two subscales of the Boredom Beliefs Scale (Tam, Van Tilburg, & Chan, 2021a). Boredom dislike subscale is a 3‐item measure assessing the extent to which participants affectively dislike boredom (e.g., “I hate being bored”; 1 = *strongly disagree*, 7 = *strongly agree*; *α* = 0.74), while boredom normalcy subscale is a 3‐item measure assessing the extent to which participants normalize the experience of boredom (e.g., “It is okay to feel bored.”; 1 = *strongly disagree*, 7 = *strongly agree*; *α* = 0.59). All the items are listed in the Supplementary Materials.

Two items were used to measure frequency (“How often have you felt bored in the last two weeks?”: 1 = *none of the time*, 9 = *all of the time*) and intensity (“When you feel bored, what is your experience of it like?”: 1 = *very mild*, 9 = *very intense*) of boredom (Tam, Van Tilburg, & Chan, 2021b).

Mental well‐being was measured with the 7‐item Short Warwick‐Edinburgh Mental Well‐being Scale (Stewart‐Brown et al., 2011). The scale focuses on positive aspects of mental health and it was validated in adolescent samples (McKay & Andretta, 2017; Ringdal et al., 2018). Participants reported what best describes their experiences over the last 2 weeks (e.g., “I've been feeling optimistic about the future”). Ratings were made on a 5‐point scale (1 = *none of the time*; 5 = *all of the time*), with higher total scores indicating more positive mental well‐being (*α* = 0.79).

#### 5.1.3 Data analysis

We examined the psychometric properties of the two boredom beliefs subscales, including their internal consistencies, factor structure, psychometric distinction from boredom experience, and measurement invariance across adolescents and young adults. To test Hypothesis 1, we examined the zero‐order correlation between boredom dislike and boredom experience. To test Hypothesis 2, we conducted regression analyses to examine whether mental well‐being was predicted by boredom dislike, boredom frequency (or boredom intensity) and their interaction terms. Simple slopes analysis was used to probe significant interactions. We also tested regression models with boredom normalcy as an exploratory predictor.

### 5.2 Results

Means, standard deviations, and correlations of the measured variables are presented in Table 1.

**TABLE 1:** Means, standard deviations, correlations of the measured variables in studies 1 and 2

	1	2	3	4	5	*M*	*SD*
1. Boredom frequency	–	0.37***	0.32***	0.10***	−0.23***	4.69	2.37
2. Boredom intensity	0.59***	–	0.33***	0.08**	−0.13***	4.74	2.26
3. Boredom dislike	0.33***	0.45***	–	0.03	−0.11***	4.16	1.63
4. Boredom normalcy	−0.01	−0.03	−0.13***	–	0.05	4.56	1.61
5. Mental well‐being	−0.36***	−0.34***	−0.20***	0.09***	–	25.47	7.08
*M*	5.60	5.10	4.38	4.87	21.81		
*SD*	2.07	2.03	1.45	1.20	4.52		

*Note*: Intercorrelations for Study 1 are presented below the diagonal, and intercorrelations for Study 2 are presented above the diagonal. Means and standard deviations for Study 1 are presented in the horizontal rows, and means and standard deviations for Study 2 are presented in the vertical columns.

** *p* < 0.01;

*** *p* < 0.001.

#### 5.2.1 Psychometric properties of boredom dislike and boredom normalcy subscales

A Confirmatory Factor Analysis (CFA) with robust maximum likelihood estimator revealed that the two‐factor model on the 6 items demonstrated fair model fit, Robust χ^2^(8) = 171.258, *p* < 0.001; Robust CFI = 0.936; Robust TLI = 0.880; Robust RMSEA = 0.096, 90% CI [0.084, 0.109]; SRMR = 0.058. Standardized factor loadings ranged from 0.55 to 0.81 for boredom dislike, and 0.29 to 0.96 for boredom normalcy. All the items loaded significantly (*p* < 0.001) on the respective factors. Boredom beliefs, boredom frequency, and boredom intensity were demonstrated to be distinct factors in the CFAs. Further, we found full configural, full metric, and partial scalar invariance, between adolescent group (below the age of 18; *n* = 1229) and adult group (at or above the age of 18; *n* = 1266). The internal consistency of boredom dislike subscale was good (*α* = 0.74; *ω* = 0.75), whereas that of boredom normalcy subscale was fair (*α* = 0.59; *ω* = 0.62). Detail results are included in Supplementary Materials.

#### 5.2.2 Relationship between boredom beliefs, boredom experience, and mental well‐being

##### Boredom dislike

Supporting Hypothesis 1, boredom dislike was positively correlated with frequency (H1a) and intensity (H1b) of boredom (Table 1). It was also negatively correlated with mental well‐being.

**FIGURE 1:**
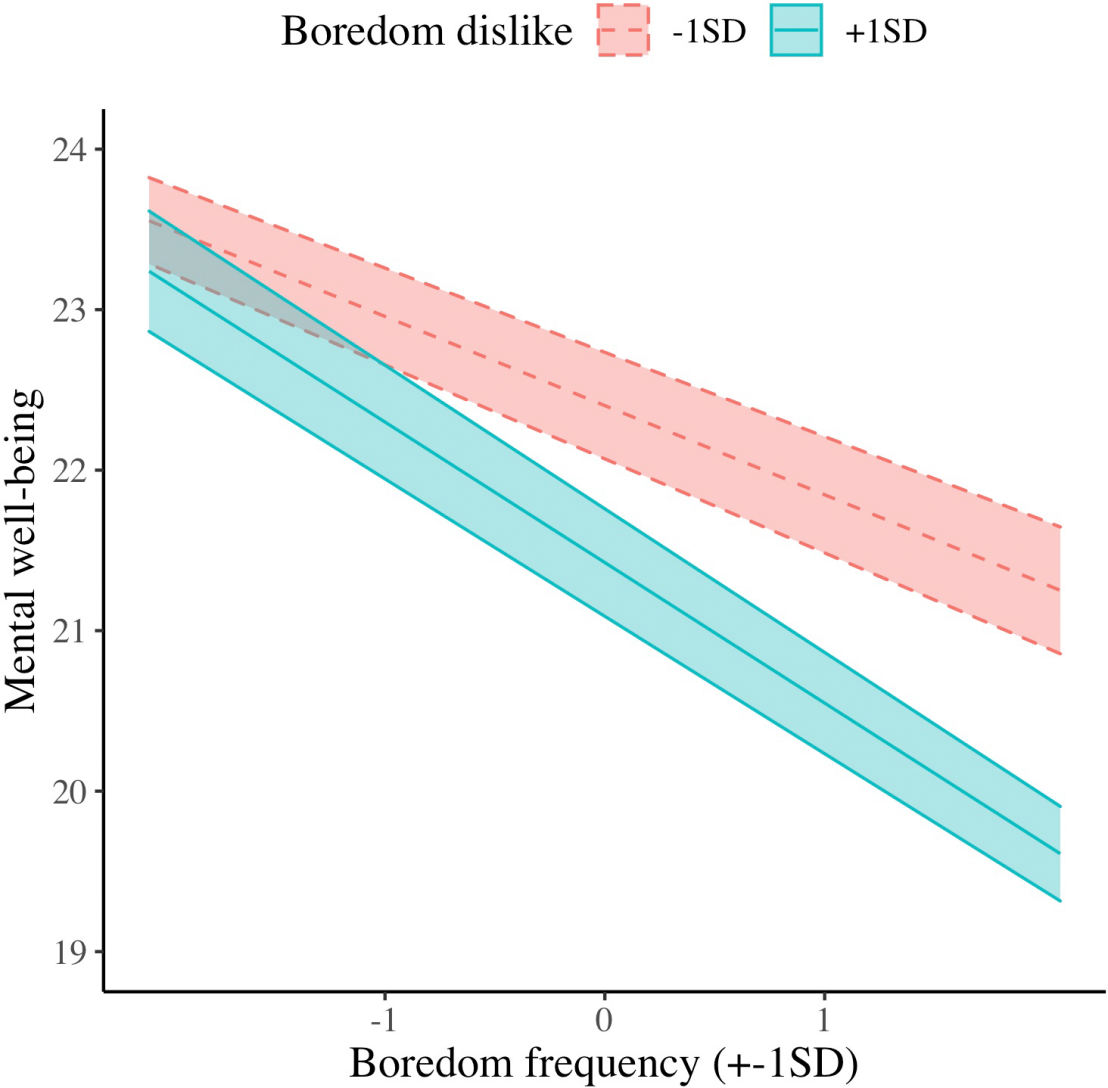
Simple slopes for between‐person associations of boredom frequency and boredom dislike in the prediction of mental well‐being in Study 1

Regarding Hypothesis 2, results of all the regression analyses are presented in Table 2. Mental well‐being was significantly associated with boredom frequency, boredom dislike, and their interaction term (H2a). Simple slopes analysis revealed that the relationship between boredom frequency and mental well‐being was significantly negative in both high (+1 SD) and low (−1 SD) levels of boredom dislike, *B* = −0.88, *SE* = 0.058, *t*(2491) = −15.1, *p* < 0.001, and *B* = −0.56, *SE* = 0.055, *t*(2491) = −10.0, *p* < 0.001 (Figure 1). These two slopes were significantly different, *B* = 0.32, *SE* = 0.075, *t*(2491) = 4.29, *p* < 0.001. A stronger association between boredom frequency and mental well‐being was found among participants who disliked boredom more. Also, we found significant main effects of boredom intensity and boredom dislike on mental well‐being, with a non‐significant boredom intensity by boredom dislike interaction (H2b).^4^

**TABLE 2:** Regression models with mental well‐being as outcome variable in Study 1

Predictor	*B*	*SE*	*β*	*p*
*Model with boredom dislike and boredom frequency*				
Intercept	21.913	0.088		
Boredom dislike	−0.337	0.062	−0.108	<0.001
Boredom frequency	−0.716	0.043	−0.328	<0.001
Boredom dislike × boredom frequency	−0.110	0.026	−0.081	<0.001
Adjusted *R* ^2^	0.140			
*Model with boredom dislike and boredom intensity*				
Intercept	21.850	0.092		
Boredom dislike	−0.189	0.066	−0.061	0.004
Boredom intensity	−0.701	0.047	−0.315	<0.001
Boredom dislike × boredom intensity	−0.033	0.027	−0.023	0.217
Adjusted *R* ^2^	0.119			
*Model with boredom normalcy and boredom frequency*				
Intercept	21.805	0.084		
Boredom normalcy	0.341	0.070	0.091	<0.001
Boredom frequency	−0.771	0.041	−0.354	<0.001
Boredom normalcy × boredom frequency	0.009	0.031	0.005	0.781
Adjusted *R* ^2^	0.133			
*Model with boredom normalcy and boredom intensity*				
Intercept	21.807	0.085		
Boredom normalcy	0.323	0.071	0.086	<0.001
Boredom intensity	−0.753	0.042	−0.339	<0.001
Boredom normalcy × boredom intensity	0.017	0.032	0.010	0.594
Adjusted *R* ^2^	0.123			

*Note*: All predictors were centered.

##### Boredom normalcy

Zero‐order correlations are presented in Table 1. Boredom normalcy was positively associated with mental well‐being, but it was not associated with boredom frequency or intensity. As shown in Table 2, regression analyses with mental well‐being as the outcome variable revealed a negative main effect of boredom frequency (or boredom intensity) and a positive main effect of boredom normalcy. There was no significant interaction between boredom frequency (or boredom intensity) and boredom normalcy.^5^

### 5.3 Discussion

In a large sample of young people aged 12–25 in the UK, we found that participants who strongly disliked boredom tended to experience it more often (H1a) and more intensely (H1b). Furthermore, participants who often felt bored were more likely to report poorer mental well‐being, but this association was weaker among those who reported a lower level of boredom dislike (H2a). The moderating effect was not observed in the association between boredom intensity and mental well‐being (H2b). Moreover, participants who accepted and normalized the experience of boredom were more likely to report better mental well‐being. The Boredom Beliefs Scale was demonstrated to be a measure with appropriate factorial validity, internal consistency, and measurement invariance across adolescents and young adults. These promising findings are, however, limited by the study's correlational design. Although they inform us of the between‐person variations in boredom beliefs (i.e., how a person differs from another in boredom beliefs), they neither speak to the malleability and stability of these beliefs, nor how within‐person variations in these beliefs (i.e., how a person differs in their levels of boredom beliefs from one occasion to another) were associated with boredom experience and mental well‐being. A better understanding of this relationship could be obtained through repeated measurements of these constructs across time and situations. As such, we conducted a second study with a multi‐wave within‐subject design to test our hypotheses.

## 6 STUDY 2

Study 2 was a repeated‐measure within‐subject study in which we assessed boredom beliefs, boredom experience, and mental well‐being among Israeli adolescents eight times across 16 weeks. In Study 1, we examined how people differ from one another in these constructs (i.e., between‐person variations); for example, we tested whether those who dislike boredom more strongly tend to feel bored more often and more intensely than others. In Study 2, we focused on how people encounter boredom from one occasion to another (i.e., within‐person variations); for example, if one's momentary boredom dislike is higher than their usual level, is that period of time characterized by higher frequency and intensity of boredom? Examining these constructs at the within‐person level helps delineate how the boredom‐health link is affected by the fluctuations in boredom beliefs within an individual.

### 6.1 Method

#### 6.1.1 Participants and procedure

Data were derived from a larger project that sought to investigate adolescents' emotional well‐being under the COVID‐19 pandemic in Israel. The study was approved by the Ethics Committee for Human Experiments at University of Haifa (ref: 368/20). Anyone aged between 12 and 18 residing in Israel at the time of data collection (from 14th May to 15th September 2020) was eligible to take part. Most participants were recruited via a survey company while some were recruited through word‐of‐mouth. They were invited to complete a baseline questionnaire and then fill out a follow‐up survey once every two weeks for seven times. A total of 498 respondents clicked on the survey link, of which 314 then consented to participate (49.0% female; age range = [12, 18], *M* = 15.5, *SD* = 1.84), with a total of 1401 data points.^6^

#### 6.1.2 Measures

We administered the same set of measures as in Study 1, namely, boredom dislike (*α* = 0.73), boredom normalcy (*α* = 0.75), boredom frequency, boredom intensity, and mental well‐being (*α* = 0.88). Measures were administered in Hebrew, after all the scales were back‐translated from English to Hebrew by two researchers who are proficient in both languages.

#### 6.1.3 Data analysis

We first attempted to replicate Study 1's results with the baseline data of Study 2. We then analyzed the multi‐wave repeated‐measure data. Multilevel modeling (MLM) was applied to account for the nested structure of the data with 1401 data points (Level 1) within 314 participants (Level 2). As all the variables were measured at Level 1, we performed within‐person centering on all the predictors to focus our analyses at the within‐person level. This procedure partitions between‐person variation (participants' scores relative to one another) in the dependent variables and the resultant level‐1 regressions represent only within‐person associations (i.e., pertaining to participants' scores at each time point relative to their own [random] means). To test Hypothesis 1, we entered boredom frequency (or boredom intensity) as the dependent variable in a multilevel model with boredom dislike as a fixed predictor, and participant as a random intercept. To test Hypothesis 2, we entered mental well‐being as the dependent variable in a multilevel model with boredom dislike, boredom frequency (or boredom intensity), and their interaction term as fixed predictors, and participant as a random intercept. Significant interactions were probed using simple slopes analyses. We conducted the same set of tests on boredom normalcy.

### 6.2 Results

#### 6.2.1 Replicating Study 1's results

Before we tested our hypotheses at within‐person level, we checked whether the Study 1's results were replicated in the Study 2's baseline data (*N* = 293). It should, however, be noted that this sample size only afforded a power of 0.80 for detecting effects sized *r* = 0.16, assuming a Type‐I error rate of 5% (two‐sided), according to sensitivity analysis. Based on the effect size of the interaction (*β* = −0.081) in Study 1, a minimum sample size of 1199, with power of 0.80, is needed to detect this effect with an alpha of 0.05.

We replicated (i) the two‐factor model in CFA, (ii) correlations between boredom dislike, boredom frequency, and boredom intensity (Hypothesis 1), as well as (iii) regression models in which mental well‐being was significantly positively associated with boredom normalcy. For Hypothesis 2, mental well‐being was significantly associated with boredom frequency but not with boredom dislike and their interaction term. This was different from Study 1, which might be attributed to the differences in sample sizes (Study 1's *N* = 2495, Study 2's *N* = 293) and thus reduced power in detecting the interaction. Detail results are included in Supplementary Materials.

#### 6.2.2 Descriptives, bivariate correlations, and intra‐class correlations

Next, we examined the within‐person associations of boredom beliefs, boredom experience, and well‐being in the multi‐wave repeated‐measure data.

Means, standard deviations, and bivariate correlations of the measured variables are presented in Table 1. In the unconditional models, the intra‐class correlations (ICCs) were 0.54 for boredom dislike, 0.52 for boredom normalcy, 0.44 for boredom frequency, 0.47 for boredom intensity, and 0.44 for mental well‐being, respectively. These values suggested considerable variability existed at the between‐person level.

#### 6.2.3 Relationship between boredom beliefs, boredom experience, and mental well‐being

##### Boredom dislike

For Hypothesis 1, boredom dislike (within‐person centered) was positively associated with boredom frequency, *B* = 0.239, *SE* = 0.048, *t*(1095) = 4.94, *p* < 0.001 (H1a), and boredom intensity, *B* = 0.282, *SE* = 0.044, *t*(1111) = 6.41, *p* < 0.001 (H1b). It was not associated with mental well‐being, *B* = 0.10, *SE* = 0.147, *t*(1107) = 0.686, *p* = 0.493.

**FIGURE 2:**
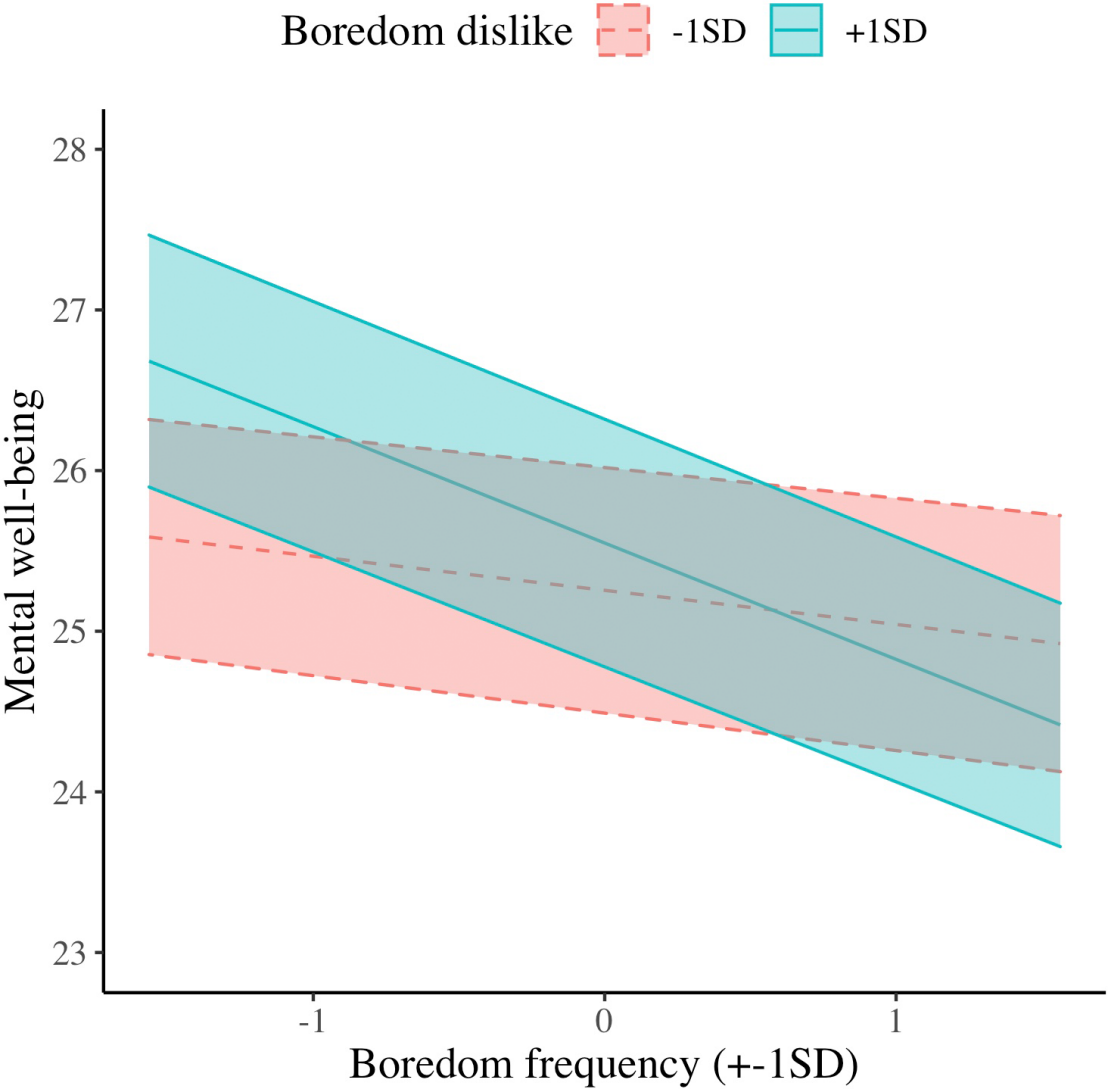
Simple slopes for within‐person associations of boredom frequency and boredom dislike in the prediction of mental well‐being in Study 2

For Hypothesis 2, results of all the random‐intercept multilevel‐modeling analyses are reported in Table 3. Mental well‐being was significantly associated with boredom frequency but not with boredom dislike. As in Study 1, the hypothesized boredom dislike × boredom frequency interaction was significant (H2a; Figure 2). Simple slopes analysis revealed that, in higher level (+1SD) of boredom dislike, the relationship between boredom frequency and mental well‐being was significant, *B* = −0.725, *SE* = 0.122, *t*(1170) = −5.925, *p* < 0.001. This relationship was not significant in lower level (−1SD), *B* = −0.212, *SE* = 0.116, *t*(1161) = −1.822, *p* = 0.069. These two slopes were significantly different, *B* = 0.512, *SE* = 0.155, *t*(1252) = 3.30, *p* = 0.001.

**TABLE 3:** Random‐intercept models with mental well‐being as outcome variable in Study 2

Predictor	*B*	*SE*	*p*	95% CI
*Model with boredom dislike and boredom frequency*				
Intercept	25.402	0.313		[24.787, 26.015]
Boredom dislike	0.152	0.147	0.301	[−0.136, 0.440]
Boredom frequency	−0.468	0.091	<0.001	[−0.646, −0.291]
Boredom dislike × boredom frequency	−0.264	0.080	<0.001	[−0.421, −0.107]
*Model with boredom dislike and boredom intensity*				
Intercept	25.406	0.314		[24.788, 26.021]
Boredom dislike	0.150	0.149	0.315	[−0.142, 0.441]
Boredom intensity	−0.156	0.100	0.121	[−0.352, 0.041]
Boredom dislike × boredom intensity	−0.207	0.088	0.019	[−0.379, −0.034]
*Model with boredom normalcy and boredom frequency*				
Intercept	25.351	0.313		[24.735, 25.964]
Boredom normalcy	0.154	0.145	0.291	[−0.131, 0.439]
Boredom frequency	−0.441	0.090	<0.001	[−0.618, −0.264]
Boredom normalcy × boredom frequency	0.033	0.081	0.687	[−0.126, 0.191]
*Model with boredom normalcy and boredom intensity*				
Intercept	25.360	0.313		[24.742, 25.971]
Boredom normalcy	0.100	0.146	0.494	[−0.186, 0.385]
Boredom intensity	−0.129	0.099	0.193	[−0.323, 0.065]
Boredom normalcy × boredom intensity	−0.004	0.093	0.963	[−0.187, 0.178]

*Note*: All predictors were within‐person centered.

**FIGURE 3:**
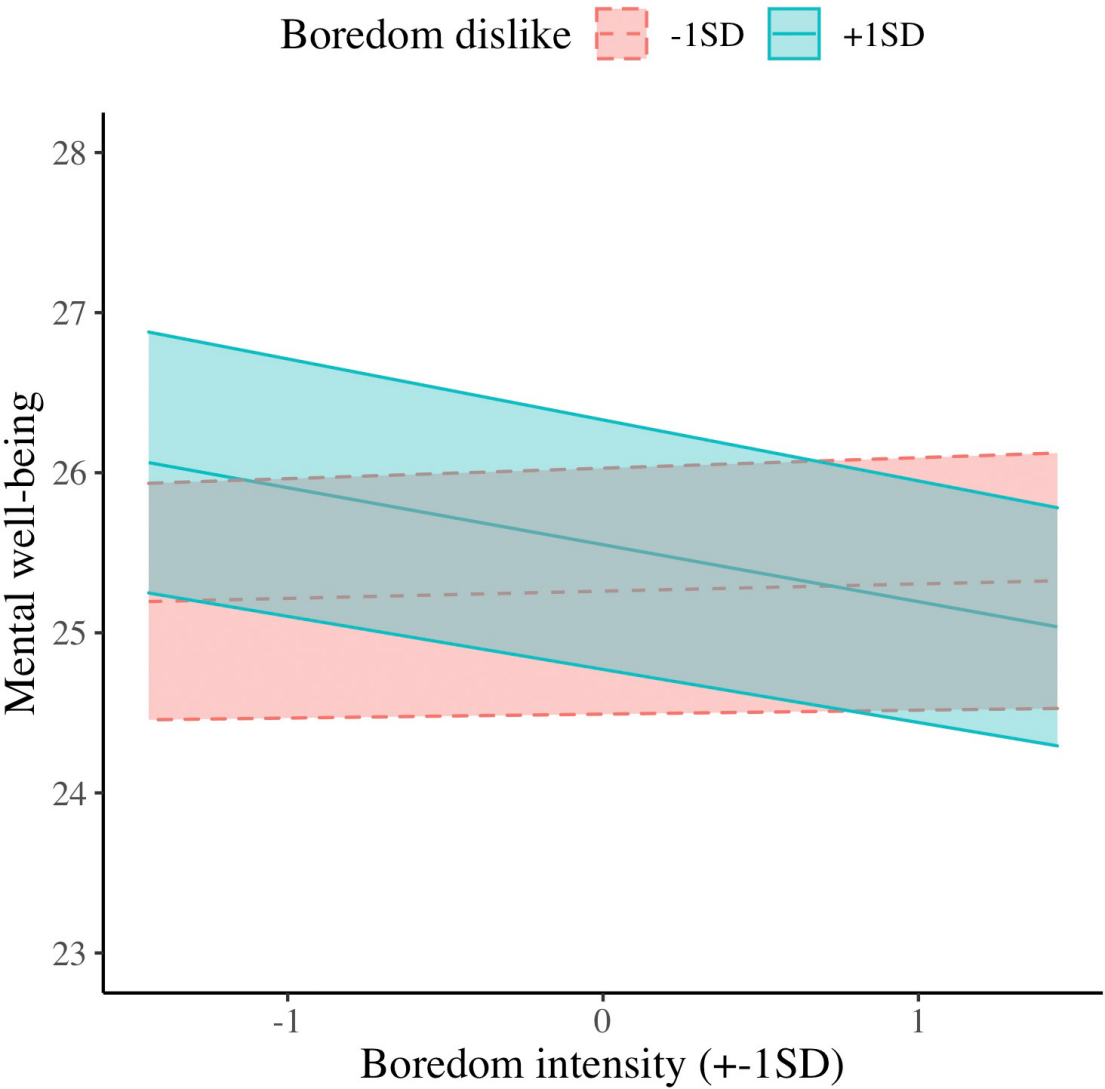
Simple slopes for within‐person associations of boredom intensity and boredom dislike in the prediction of mental well‐being in Study 2

Moreover, when mental well‐being was the outcome variable, the main effects of boredom dislike and boredom intensity were not significant. Unlike in Study 1, the hypothesized boredom dislike × boredom intensity interaction was significant (H2b; Figure 3). Simple slopes analysis revealed that, in higher level (+1SD) of boredom dislike, the relationship between boredom intensity and mental well‐being was significant, *B* = −0.356, *SE* = 0.136, *t*(1157) = −2.628, *p* = 0.009. This relationship was not significant in lower level (−1SD), *B* = 0.045, *SE* = 0.128, *t*(1148) = 0.353, *p* = 0.724. These two slopes were significantly different, *B* = 0.401, *SE* = 0.171, *t*(1224) = 2.35, *p* = 0.019.

##### Boredom normalcy

Boredom normalcy (within‐person centered) was not significantly associated with mental well‐being, *B* = 0.085, *SE* = 0.145, *t*(1107) = 0.586, *p* = 0.558. It was positively associated with boredom frequency, *B* = 0.139, *SE* = 0.048, *t*(1096) = 2.87, *p* = 0.004, and boredom intensity, *B* = 0.111, *SE* = 0.044, *t*(1112) = 2.50, *p* = 0.013.

As shown in Table 3, multilevel analysis with mental well‐being as the outcome variable revealed a significant main effect of boredom frequency, with a non‐significant main effect of boredom normalcy and a non‐significant boredom normalcy × boredom frequency interaction. Moreover, there was no significant main effect of boredom intensity, boredom normalcy, and their interaction on mental well‐being.

### 6.3 DISCUSSION

Study 2's results are similar to those in Study 1. Using multi‐wave repeated‐measure data from Israeli adolescents aged 12–18, multilevel modeling analyses revealed that participants experienced boredom more frequently (H1a) and intensely (H1b) when they disliked boredom more strongly than their own average fortnight. Furthermore, participants reported poorer mental well‐being when they felt bored more often and more intensely; these associations were stronger at times they reported a higher level of boredom dislike (H2a & H2b).

The results on boredom normalcy were less consistent with Study 1. While Study 1 showed a significant positive association between boredom normalcy and mental well‐being, this association was not significant in Study 2. Because between‐person associations cannot, and should not, be used to make assertions about within‐person associations (Snijders & Bosker, 2011; Walker, 2021), a possible explanation is that the relationship of these variables was different at within‐person level (i.e., whether participants reported better mental well‐being *at times* they normalized boredom more than their usual) than at the between‐person level (i.e., whether participants who normalized boredom *more than others* reported better mental well‐being than other participants). Indeed, our analysis with the baseline data revealed a positive association between boredom normalcy and mental well‐being at between‐person level (reported in Supplementary Materials), which replicated the findings from Study 1. In other words, people who report higher boredom normalcy than others are more likely to report better mental well‐being than other people; however, when people momentarily normalize boredom more, those periods are not characterized by better mental well‐being.

## 7 GENERAL DISCUSSION

Chronic boredom can lead to problematic behaviors (e.g., Biolcati et al., 2018; Malizia, 2018) and psychological distress (Spaeth et al., 2015) among young people. Given that young people are more prone to boredom (Caldwell et al., 1999; Weybright et al., 2020), boredom may be particularly challenging for them during the COVID‐19 pandemic. Across two studies, we examined the moderating effect of boredom beliefs on the boredom‐mental well‐being link among adolescents and young adults in the UK and Israel. The results consistently demonstrate that, at both between‐ (Study 1) and within‐person levels (Study 2), disliking boredom was positively associated with frequency (H1a) and intensity of boredom (H1b). Also, boredom dislike moderated the negative associations of boredom frequency (Studies 1 and 2) and boredom intensity (Study 2) with mental well‐being. Specifically, the associations were stronger in higher level of boredom dislike (H2a & H2b). In addition, participants who relatively accepted and normalized boredom reported better mental well‐being.

We validated two subscales of the Boredom Beliefs Scale (Tam, Van Tilburg, & Chan, 2021a) in a sample of young people. The reliability and validity of the subscales were comparable to those of the Hong Kong and the US samples reported in Tam, Van Tilburg, and Chan (2021a). The 6‐item measure replicated the two‐factor structure, and was shown to be distinct from boredom experience in CFAs. The current results demonstrated full configural, full metric, and partial scalar invariance across the two age groups (adolescent and young adult), indicating that the factor structure fit well and that factor loadings are similar across these age groups. The achievement of full metric invariance suggests that adolescents and young adults responded to the items similarly (Steenkamp & Baumgartner, 1998). Failure to find scalar invariance indicates that mean differences of item responses are not the same as the mean differences in the latent variables (Putnick & Bornstein, 2016). Caution should thus be made when directly comparing the mean scores across age groups. In terms of test–retest reliability, the ICCs of boredom beliefs were comparable to those of emotion beliefs in previous research (Veilleux et al., 2021).

Our results supported Hypothesis 1. Higher levels of boredom dislike, relative to others and relative to one's average level, were associated with higher levels of boredom frequency and intensity. This aligns with earlier findings on a positive association found between boredom dislike and boredom proneness (Tam, Van Tilburg, & Chan, 2021a) and those on an inverse association between “liking” withdrawal emotion and the intensity of emotional experience (Harmon‐Jones et al., 2011). Boredom normalcy was not significantly associated with boredom frequency and intensity at the between‐person level, which is consistent with the results on the non‐significant association between boredom normalcy and boredom proneness (Tam, Van Tilburg, & Chan, 2021a). It was, however, positively associated with boredom frequency and intensity at the within‐person level, which indicates that people are more inclined to believe that boredom is a normal experience at times they feel bored more frequently and intensely.

For Hypothesis 2, the present research demonstrated a moderating effect of boredom dislike with boredom frequency (and boredom intensity in Study 2) in predicting mental well‐being. It indicates that young people who felt bored more often reported a lower level of mental well‐being; this relationship was weaker among those who held a more positive affective evaluation of boredom. This result parallels those on the moderating effect of negative affect valuation on the linkage between negative affective experiences and well‐being (Luong et al., 2016). Taking a step further, we also examined the moderating effect at the within‐person level. We found that when young people disliked boredom more than their average level, the negative associations of boredom frequency and intensity with mental well‐being were stronger.

The associations of mental well‐being with boredom dislike and boredom normalcy were significant at the between‐person level, but not significant at the within‐person level. At the between‐person level (i.e., compared with other participants), higher levels of (affective) disliking and (cognitive) unacceptance of boredom were linked with poorer well‐being, as revealed in the bivariate correlation and in regression models controlling for boredom frequency or intensity in Study 1. This is consistent with a study that reported a positive relationship between negative attitudes toward emotion and depressive symptoms (Yoon et al., 2018). At the within‐person level, the associations of mental well‐being with boredom dislike and boredom normalcy were nonsignificant. This suggests that mental well‐being is not linked with within‐person fluctuation in levels of boredom dislike and boredom normalcy.

Boredom functions to signal a need for behavioral change (e.g., Bench & Lench, 2019; Danckert et al., 2018; Wolff & Martarelli, 2020). It is possible that people who hate boredom or do not normalize the experience are less able to respond to it adaptively. They might thus (i) evaluate their boredom experience more negatively, (ii) without knowing how to regulate it in an effective or adaptive manner. These might, in turn, make their experience more unpleasant and influence their mental well‐being (Ford & Gross, 2019). This potential mediating role of emotion regulation warrants future investigation.

### 7.1 Strengths and limitations

The present research is comprised of a correlational study with British young people and a multi‐wave within‐subject study with Israeli adolescents. The replication of findings using different methods at both between‐ and within‐person levels in two different countries offers strong support to the generalizability of the results. Large sample sizes and ecological validity are other key strengths. Yet, the findings should be interpreted with the consideration of several limitations. First, given the correlational nature of the findings, the results cannot establish causality between the measured variables. For example, the relationship between boredom beliefs and boredom experience could be bidirectional. It is possible that people dislike boredom because they feel it very often with high intensity, or they more readily pick up the cues of boredom and feel it frequently and intensely because they strongly dislike this emotion. Future studies using an experimental approach would be helpful in elucidating their relationships. Second, we did not administer the full version of the Boredom Beliefs Scale; we omitted the boredom functionality subscale. This was because our studies were part of a larger project that involved several research teams with different research focuses. To keep the biweekly survey within a reasonable length, we could not include the 9 items on boredom functionality. We chose to include boredom dislike and boredom normalcy because (i) they appear to be most similar to the emotion beliefs on valuing and accepting emotion in the literature (Harmon‐Jones et al., 2011; Luong et al., 2016), and (ii) these two subscales are relatively short (6 items in total). Future research is needed to examine how the boredom functionality subscale performs in youth samples. Third, we failed to find scalar invariance for the two subscales across age groups and the boredom normalcy subscale's internal consistency appeared low in Study 1. One possible reason is that the scale was developed from Hong Kong and American samples, and thus performed poorer in British sample. Future research is required to examine their psychometric properties across cultural and age groups.

### 7.2 Implications

To the best of our knowledge, this is the first study that investigated youth boredom beliefs and among the first to study boredom beliefs in general. It was conducted during the COVID‐19 pandemic—a high‐risk context in which boredom was especially difficult to escape. Accumulating studies have pointed to the undesirable effects of boredom in the pandemic (e.g., Boylan et al., 2021; Chao et al., 2020; Wolff et al., 2020); yet, limited research has examined how they can be ameliorated. Our studies contribute to the literature by presenting timely, promising findings on the role of boredom beliefs in altering the mental health impact of boredom. It offers novel insights on potential intervention and preparation for similar high‐risk situations in the future. The considerable within‐person variability in boredom beliefs shown in Study 2 suggests that these beliefs fluctuate over time and thus they could be the target of intervention. Future research could investigate, for example, if education on the values of boredom reduces young people's boredom dislike and promotes their well‐being.

Researchers (Martarelli & Wolff, 2020) argue that the pandemic containment policies of restricted social, educational, and recreational activities likely intensify boredom and impose self‐control demands that are particularly challenging for young people. Considering the findings on boredom and non‐compliance to pandemic measures (Boylan et al., 2021; Brosowsky et al., 2021; Wolff et al., 2020), and that young people are poorer at self‐control (Casey & Caudle, 2013), they might be at higher risk of engaging in impulsive, problematic behaviors in response to boredom during the pandemic. Future research can consider examining the role of boredom beliefs in these relationships.

As this is the first study that examined lay beliefs about boredom in an adolescent sample, it raised more questions than it answered, such as why some young people hate boredom more than others and how to intervene on boredom dislike. These questions could be investigated in future studies using the boredom dislike and boredom normalcy subscales we validated in the present research. For instance, the measures could be applied in educational contexts—where detrimental effects of boredom on academic performance are well documented (Tze et al., 2016)—to understand the role of boredom beliefs.

## 8 CONCLUSION

The detrimental mental health impact of chronic boredom is evident, and the COVID‐19 pandemic might have worsened it. The present correlational and multi‐wave within‐subject studies demonstrated that disliking boredom is associated with more frequent and intense boredom experiences. The negative association between boredom and mental well‐being is more salient if young people dislike this emotion strongly. Normalizing the occurrence of boredom, on the contrary, is associated with better mental well‐being. Additionally, we validated a measure of boredom beliefs in two youth samples. Overall, this research underscored the importance of boredom beliefs on boredom experience and mental well‐being.

## AUTHOR CONTRIBUTIONS

Katy Y. Y. Tam: Conceptualization, Data Analysis, Writing – Original Draft Preparation, Writing – Review & Editing. Christian S. Chan: Writing – Review & Editing, Supervision. Wijnand A. P. van Tilburg: Writing – Review & Editing, Supervision. Iris Lavi: Funding Acquisition, Project Administration, Writing – Review & Editing. Jennifer Y. F. Lau: Conceptualization, Funding Acquisition, Methodology, Project Administration, Writing – Review & Editing, Supervision.

## CONFLICT OF INTEREST

The authors have no conflict of interest to declare.

## ETHICS STATEMENT

The data were collected in accordance with the ethical standards of APA. This research was approved by the Psychiatry, Nursing and Midwifery Research Ethics Committee at Kings College London (ref: HR‐19/20‐18868), and the Ethics Committee for Human Experiments at University of Haifa (ref: 368/20).

## Supporting information


Appendix S1

## Endnotes

^1^
We examined the pattern of missing data in this sample (*N* = 2560) and the amount of missing data at item level was 1.84%. We further ran regression analyses using the method of replacement of missing values by mean; results stayed largely the same. Detailed results are reported in the Supplementary Materials.

^2^
Main results of Studies 1 and 2 with gender as covariate are presented in the Supplementary Materials. They were similar to what we found without controlling for gender.

^3^
There were no missing data in this sample (*N* = 2495).

^4^
An item “Boredom drags down my mood” in the boredom dislike subscale might potentially conflate with mental well‐being. To check whether this item disproportionately affected our results in both Studies 1 and 2, we conducted the same analyses with a composite of boredom dislike excluding this item. Results stayed largely the same and they are presented in the Supplementary Materials.

^5^
We conducted separate analyses for boredom dislike and normalcy, and for boredom frequency and intensity given their conceptual distinction. Result with all these predictors in the same model stayed largely the same, but it raises some challenging questions such as what boredom frequency and boredom intensity mean when their shared variance are partialled out; what the interaction term means when the other interaction term is controlled for. Therefore, we retained our analyses that featured separate models in the main text and presented these additional analyses in the Supplementary Materials.

^6^
We examined the pattern of missing data in this sample (*N =* 314), and the amount of missing data at item level was 44.0% (detailed results presented in the Supplementary Materials). The high level of missingness was attributed to dropouts over the course of the eight‐wave longitudinal study. At any given time point, participants either responded to all the items of the key variables (i.e., items for assessing boredom frequency, boredom intensity, boredom dislike, boredom normalcy and mental well‐being) or responded to none of them. As such, imputation was not performed to handle the missing data.
