# Supplementary material for: Boredom belief moderates the mental health impact of boredom among young people: Correlational and multi‐wave longitudinal evidence gathered during the COVID‐19 pandemic
Source: J Pers. 2022 Aug 21:10.1111/jopy.12764. Online ahead of print. doi: 10.1111/jopy.12764 (PMC9537911; doi:10.1111/jopy.12764)
Supplement: Supplementary file 1 — Appendix S1 [file JOPY-9999-0-s001.docx]

**Supplementary Materials**

Boredom belief moderates the mental health impact of boredom among young people: Correlational and multi-wave longitudinal evidence gathered during the COVID-19 pandemic

Katy Y. Y. Tam

The University of Hong Kong and King’s College London

Christian S. Chan

The University of Hong Kong

Wijnand A. P. van Tilburg

University of Essex

Iris Lavi

University of Bath and University of Haifa

Jennifer Y. F. Lau

Queen Mary University of London

Table of Contents

[Missing Data Analyses in Studies 1 and 2 3](#_Toc108793599)

[Validation of the Boredom Beliefs Scale in Studies 1 and 2 5](#_Toc108793600)

[Results Controlling for Gender in Studies 1 and 2 12](#_Toc108793601)

[Testing the Influence of an Item in Boredom Dislike Subscale on Main Results in Studies 1 and 2 14](#_Toc108793602)

[Testing a Regression Model with All the Predictors in Study 1 15](#_Toc108793603)

[Testing the Between-person Associations of Boredom Beliefs, Boredom Experience and Mental Well-being in Study 2 16](#_Toc108793604)

[References 18](#_Toc108793605)

# Missing Data Analyses in Studies 1 and 2

Surveys in both studies were designed in a way that respondents could not skip questions (i.e., they had to submit a response before proceeding to the next question). Any missing data, therefore, came from participants discontinuing the survey (i.e. closing the browser down) or discontinuing the study (i.e. not completing subsequent surveys, in the case of Study 2, the multi-wave study). In Study 1, we excluded participants (*n* = 65) who had missing data on any of the key variables (i.e., boredom frequency, boredom intensity, boredom dislike, boredom normalcy and mental well-being), which resulted in the final sample of 2,495. We conducted a missing data analysis on the sample including the missing data (*N* = 2,560), and the results are presented in Table S1. The amount of missing data at item level was 1.84%. We conducted regression analyses using the method of replacement of missing values by mean. Results stayed largely the same as those in the main manuscript (Table S2).

**Table S1**

*Pattern of Missing Data in Studies 1 and 2*

|  | Study 1 (*N* = 2560, 1 wave) | |  | Study 2 (*N* = 314, 8 waves) | |
| --- | --- | --- | --- | --- | --- |
| Item | No. of responses | No. of missing responses (%) |  | No. of responses | No. of missing responses (%) |
| Boredom frequency | 2499 | 61 (2.4%) |  | 1401 | 1111 (44.2%) |
| Boredom intensity | 2499 | 61 (2.4%) |  | 1401 | 1111 (44.2%) |
| BBS item 1 | 2495 | 65 (2.5%) |  | 1401 | 1111 (44.2%) |
| BBS item 2 | 2495 | 65 (2.5%) |  | 1401 | 1111 (44.2%) |
| BBS item 3 | 2495 | 65 (2.5%) |  | 1401 | 1111 (44.2%) |
| BBS item 4 | 2495 | 65 (2.5%) |  | 1401 | 1111 (44.2%) |
| BBS item 5 | 2495 | 65 (2.5%) |  | 1401 | 1111 (44.2%) |
| BBS item 6 | 2495 | 65 (2.5%) |  | 1401 | 1111 (44.2%) |
| Well-being item 1 | 2532 | 28 (1.1%) |  | 1414 | 1098 (43.7%) |
| Well-being item 2 | 2532 | 28 (1.1%) |  | 1414 | 1098 (43.7%) |
| Well-being item 3 | 2532 | 28 (1.1%) |  | 1414 | 1098 (43.7%) |
| Well-being item 4 | 2532 | 28 (1.1%) |  | 1414 | 1098 (43.7%) |
| Well-being item 5 | 2532 | 28 (1.1%) |  | 1414 | 1098 (43.7%) |
| Well-being item 6 | 2532 | 28 (1.1%) |  | 1414 | 1098 (43.7%) |
| Well-being item 7 | 2532 | 28 (1.1%) |  | 1414 | 1098 (43.7%) |

**Table S2**

*Regression Models with Mental Well-being as Outcome Variable in Study 1*

| Predictor | *B* | *SE* | β | *p* |
| --- | --- | --- | --- | --- |
| **Model with boredom dislike and boredom frequency** | | | | |
| Intercept | 21.898 | 0.087 |  |  |
| Boredom dislike | -0.337 | 0.062 | -.108 | < .001 |
| Boredom frequency | -0.715 | 0.043 | -.326 | < .001 |
| Boredom dislike × boredom frequency | -0.110 | 0.026 | -.081 | < .001 |
| *Adjusted R^2^* | .138 |  |  |  |
| **Model with boredom dislike and boredom intensity** | | | | |
| Intercept | 21.835 | 0.092 |  |  |
| Boredom dislike | -0.189 | 0.066 | -.060 | .004 |
| Boredom intensity | -0.701 | 0.047 | -.313 | < .001 |
| Boredom dislike × boredom intensity | -0.033 | 0.027 | -.023 | .217 |
| *Adjusted R^2^* | .117 |  |  |  |
| **Model with boredom normalcy and boredom frequency** | | | | |
| Intercept | 21.790 | 0.084 |  |  |
| Boredom normalcy | 0.341 | 0.070 | .090 | < .001 |
| Boredom frequency | -0.771 | 0.041 | -.352 | < .001 |
| Boredom normalcy × boredom frequency | 0.009 | 0.031 | .005 | .781 |
| *Adjusted R^2^* | .132 |  |  |  |
| **Model with boredom normalcy and boredom intensity** | | | | |
| Intercept | 21.791 | 0.084 |  |  |
| Boredom normalcy | 0.323 | 0.071 | .085 | < .001 |
| Boredom intensity | -0.753 | 0.042 | -.337 | < .001 |
| Boredom normalcy × boredom intensity | 0.017 | 0.032 | .010 | .594 |
| *Adjusted R^2^* | .121 |  |  |  |

*Note.* All predictors were centered. Regression analyses were performed using the method of replacement of missing values by mean.

In Study 2, there were 184 participants who clicked the link but did not provide any data. Among those who actually participated (*N* = 314), some had missing data in this sample due to intermitting or dropping out entirely from the eight-wave longitudinal study (see Table S1). At any given time point, participants either responded to all the items of the key variables (i.e., items for assessing boredom frequency, boredom intensity, boredom dislike, boredom normalcy and mental well-being) or responded to none of them (i.e. they did not contribute to an entire wave). As such, imputation was not performed to handle the missing data.

# Validation of the Boredom Beliefs Scale in Studies 1 and 2

Given that the boredom dislike and boredom normalcy subscales have not been validated in the UK or in adolescent samples, we examined their psychometric properties, including their factor structure (Studies 1 and 2), whether they were psychometrically distinct from boredom experience (Study 1), and their measurement invariance across age groups (Study 1).

**Factor Analysis**

***Data Analysis***

We conducted a confirmatory factor analysis (CFA) to validate the two-factor structure on the six items in both studies. Robust comparative fit index (CFI) and robust Tucker–Lewis index (TLI) greater than .90, robust root mean squared error of approximation (RMSEA) and standardized root mean squared residual (SRMR) values less than .08 are indicative of good fit (Hu & Bentler, 1999).

***Results***

In Study 1’s UK sample (*N* = 2,495), the two-factor model demonstrated fair model fit, Robust χ^2^(8) = 171.258, *p* < .001; Robust CFI = .936; Robust TLI = .880; Robust RMSEA = .096, 90% CI [.084, .109]; SRMR = .058. Standardized factor loadings ranged from .55 to .81 for boredom dislike, and .29 to .96 for boredom normalcy (Table S3). All the items loaded significantly (*p* < .001) on the respective factors. We compared the two-factor model with a single factor model; the single factor model was significantly poorer fitting, Δχ^2^ = 434.42, *p* < .001; Δ Robust CFI = .258; Δ Robust TLI = .417, Δ Robust RMSEA = -.108, Δ SRMR = -.070.

We ran the same CFA with the baseline data of Study 2’s Israel sample (*N* = 293). The two-factor model demonstrated fair model fit, Robust χ^2^(8) = 27.22, *p* = .001; Robust CFI = .936; Robust TLI = .881; Robust RMSEA = .097, 90% CI [.058, .138]; SRMR = .078. Standardized factor loadings ranged from .46 to .84 for boredom dislike, and .48 to .70 for boredom normalcy (Table S3). All the items loaded significantly (*p* < .001) on the respective factors. We compared the two-factor model with a single factor model; the single factor model was significantly poorer fitting, Δχ^2^ = 118.41, *p* < .001; Δ Robust CFI = .313; Δ Robust TLI = .510, Δ Robust RMSEA = -.125, Δ SRMR = -.077.

**Table S3**

*Standardized Factor Loadings from Confirmatory Factor Analysis of the 6 Items*

| Item | | Study 1 | |  | Study 2 | |
| --- | --- | --- | --- | --- | --- | --- |
|  |  | Boredom dislike | Boredom normalcy |  | Boredom dislike | Boredom normalcy |
| 2 | I am afraid of being bored | .55 |  |  | .46 |  |
| 3 | I hate being bored | .75 |  |  | .76 |  |
| 4 | Boredom drags down my mood | .81 |  |  | .84 |  |
| 1 | Sometimes people have to learn to live with boredom |  | .29 |  |  | .48 |
| 5 | Boredom is a natural emotional response |  | .47 |  |  | .70 |
| 6 | It is okay to feel bored |  | .96 |  |  | .67 |

**Psychometric Distinction between Boredom Beliefs and Boredom Experience**

***Data Analysis***

We conducted factor analyses with four different models (Figure S1), and compared these models to examine whether boredom beliefs can be measured distinctly from boredom frequency and intensity. CFAs with robust maximum likelihood estimator were conducted with the specifications of the following factor structures: (a) *Four-factor model* in which boredom dislike items, boredom normalcy items, boredom frequency item, and boredom intensity item were loaded on four respective factors; (b) *One-factor model* in which all the items of boredom dislike, boredom normalcy, boredom frequency and boredom intensity were loaded on a single factor; (c) *Two-factor model (boredom dislike)* in which boredom dislike items, boredom frequency item and boredom intensity item were loaded on a factor, while boredom normalcy items loaded on another factor; (d) *Two-factor model (boredom normalcy)* in which boredom normalcy items, boredom frequency item and boredom intensity item were loaded on a factor, while boredom dislike items loaded on another.

**Figure S1**

*Conceptual CFA Models*

**
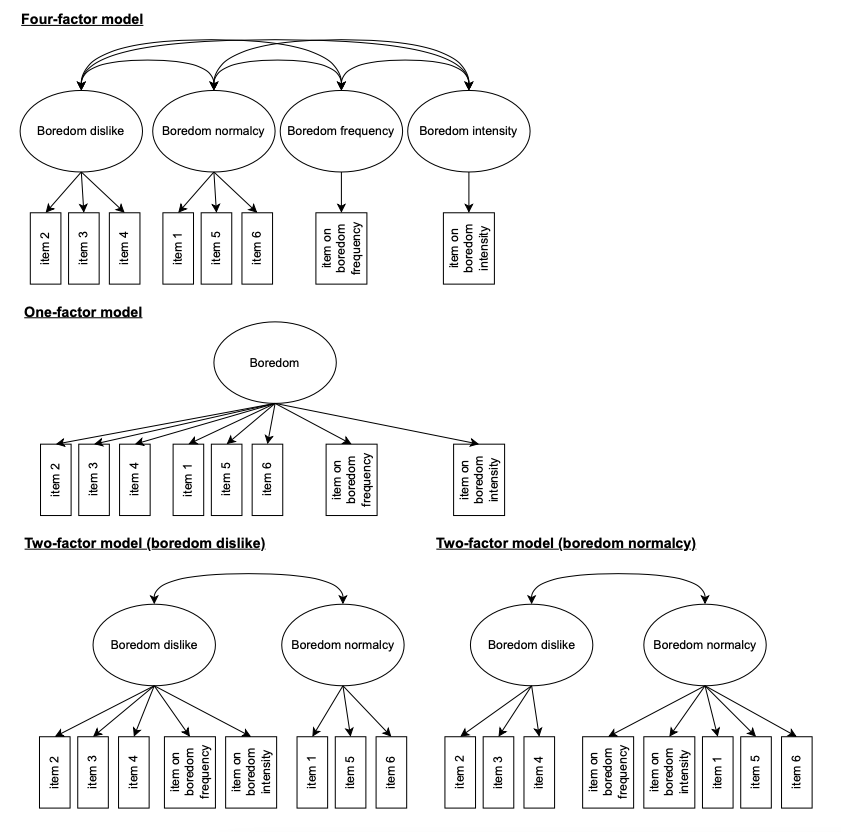
**

***Results***

Model fit indices from CFAs and results from chi-square difference test comparing the models that are nested are reported in Table S4 and Table S5. The four-factor model was shown to be the best-fitting model. These results suggest that boredom beliefs and boredom experience are psychometrically distinct.

**Table S4**

*Model Fit Indices*

| Model | Robust χ^2^ | Robust CFI | Robust TLI | Robust RMSEA [90 % CI] | SRMR |
| --- | --- | --- | --- | --- | --- |
| Four-factor model | 200.61 | .955 | .921 | .072 [.064, .081] | .051 |
| One-factor model | 1357.494 | .656 | .519 | .178 [.170, .186] | .115 |
| Two-factor model (boredom dislike) | 725.575 | .821 | .736 | .132 [.124, .140] | .077 |
| Two-factor model (boredom normalcy) | 1596.106 | .603 | .415 | .196 [.188, .205] | .163 |

**Table S5**

*Chi-square Difference Test Between Models*

| Model | *df* | *AIC* | *BIC* | χ^2^ | Δ χ^2^ | Δ*df* | *p* |
| --- | --- | --- | --- | --- | --- | --- | --- |
| Four-factor model | 16 | 75346 | 75463 | 227.07 |  |  |  |
| Two-factor model (boredom dislike) | 19 | 75960 | 76059 | 847.01 | 456.98 | 3 | < .001 |
| Two-factor model (boredom normalcy) | 19 | 76965 | 77064 | 1851.46 | 1004.45 | 0 |  |
| One-factor model | 20 | 76719 | 76812 | 1607.62 | -148.19 | 1 | 1 |

**Measurement Invariance**

***Data Analysis***

We then conducted multigroup CFA to test the measurement invariance of the scale across age groups in Study 1. The total sample was split at the age of 18, which is the legal age of adulthood in the UK. This yielded an adolescent group (below the age of 18; *n* = 1,229) and an adult group (at or above the age of 18; *n* = 1,266). We examined a configural model, in which all parameters were freely estimated, to test whether the factor structure was significantly invariant across the two age groups (i.e., configural invariance model); we then estimated metric invariance by constraining factor loadings to be equal across the two groups, and we further tested scalar invariance by constraining the item intercepts to be equal. In stepwise approach, the metric invariance model was compared against the configural invariance model; the scalar invariance model was compared against the metric invariance model. If the change of CFI (ΔCFI) does not exceed .010 (Cheung & Rensvold, 2002) and the RMSEA value falls within the comparing model’s RMSEA confidence intervals (Timmons, 2010), invariance is established.

***Results***

Table S6 presents the fit indices for the measurement invariance of the two-factor model. The fit of the configural invariance model was fair, χ^2^(16) = 193.99, CFI = .940, RMSEA = .094, SRMR = .051. It suggests that the overall factor structure fits well across the two age groups. Constraining the factor loadings to be equal resulted in minor fit deterioration, with ΔCFI = .001 and RMSEA value falling within the RMSEA confidence intervals of the configural invariance model. Metric invariance was thus supported. Applying further restrictions with respect to the item intercepts, however, resulted in significant worsening in model fit, where ΔCFI = .041 and RMSEA value fall outside of the RMSEA confidence intervals of the metric invariance model. Scalar invariance was not supported. Following the suggestions by Putnick and Bornstein (2016), we investigated partial invariance by releasing item intercept constraints to one item per latent constructs (items 1 and 2). We then compared the partially invariant model with metric invariant model, which showed that ΔCFI = .007 and RMSEA value fell outside of the RMSEA confidence intervals of the metric invariance model. Partial scalar invariance was found. Taken together, we found full configural invariance, full metric invariance and partial scalar invariance across the two age groups.

**Table S6**

*Model Fit Indices for the Measurement Invariance Models of the Two Age Groups*

|  |  |  |  |  | Difference tests | | | | |
| --- | --- | --- | --- | --- | --- | --- | --- | --- | --- |
| Model | χ^2^ | *df* | CFI | RMSEA [90% *CI*] | ΔCFI | ΔRMSEA | Δχ^2^ | Δ*df* | *p* |
| Configural invariance | 193.99 | 16 | .940 | .094 [.083, .107] |  |  |  |  |  |
| Metric invariance | 200.08 | 20 | .939 | .085 [.074, .096] | -.001^a^ | -.009 | 6.09 | 4 | .193 |
| Scalar invariance | 324.50 | 24 | .898 | .100 [.091, .110] | -.041^b^ | .015 | 124.42 | 4 | < .001 |
| Partial scalar invariance | 233.32 | 22 | .932 | .086 [.076, .096] | -.007^c^ | .001 | 23.239 | 2 | < .001 |

*Note. df* = degrees of freedom; CFI = comparative fit index; RMSEA = root mean square error of approximation; 90% *CI* = 90% confidence interval.

^a^Difference between configural and metric invariance models.

^b^Difference between metric and scalar invariance models.

^c^Difference between metric and partial scalar invariance models.

# Results Controlling for Gender in Studies 1 and 2

We tested linear regression models (Study 1) and random-intercept models (Study 2) controlling for gender as exploratory analyses. Results are presented in Table S7 and Table S8. They were similar to what we found without controlling for gender.

**Table S7**

*Regression Models with Mental Well-being as Outcome Variable in Study 1*

| Predictor | *B* | *SE* | β | *p* |
| --- | --- | --- | --- | --- |
| **Model with boredom dislike and boredom frequency** | | | | |
| Intercept | 23.042 | 0.153 |  |  |
| Gender (-1=male, 1=female) | -1.618 | 0.181 | -0.164 | < .001 |
| Boredom dislike | -0.331 | 0.061 | -0.106 | < .001 |
| Boredom frequency | -0.689 | 0.042 | -0.316 | < .001 |
| Boredom dislike × boredom frequency | -0.104 | 0.025 | -0.077 | < .001 |
| *Adjusted R^2^* | 0.166 |  |  |  |
| **Model with boredom dislike and boredom intensity** | | | | |
| Intercept | 23.052 | 0.157 |  |  |
| Gender (-1=male, 1=female) | -1.716 | 0.183 | -0.174 | < .001 |
| Boredom dislike | -0.184 | 0.065 | -0.059 | .005 |
| Boredom intensity | -0.682 | 0.046 | -0.307 | < .001 |
| Boredom dislike × boredom intensity | -0.031 | 0.027 | -0.022 | .242 |
| *Adjusted R^2^* | 0.148 |  |  |  |
| **Model with boredom normalcy and boredom frequency** | | | | |
| Intercept | 22.940 | 0.152 |  |  |
| Gender (-1=male, 1=female) | -1.618 | 0.182 | -0.164 | < .001 |
| Boredom normalcy | 0.322 | 0.069 | 0.086 | < .001 |
| Boredom frequency | -0.745 | 0.040 | -0.342 | < .001 |
| Boredom normalcy × boredom frequency | 0.007 | 0.031 | 0.004 | .820 |
| *Adjusted R^2^* | 0.160 |  |  |  |
| **Model with boredom normalcy and boredom intensity** | | | | |
| Intercept | 22.997 | 0.153 |  |  |
| Gender (-1=male, 1=female) | -1.697 | 0.182 | -0.172 | < .001 |
| Boredom normalcy | 0.302 | 0.070 | 0.080 | < .001 |
| Boredom intensity | -0.733 | 0.041 | -0.330 | < .001 |
| Boredom normalcy × boredom intensity | 0.013 | 0.031 | 0.008 | .688 |
| *Adjusted R^2^* | 0.152 |  |  |  |

*Note.* All continuous predictors were centered.

**Table S8**

*Random-intercept Models with Mental Well-being as Outcome Variable in Study 2*

| Predictor | *B* | *SE* | *p* | *95% CI* |
| --- | --- | --- | --- | --- |
| **Model with boredom dislike and boredom frequency** | | | | |
| Intercept | 25.925 | 0.438 |  | [25.066, 26.781] |
| Gender (-1=male, 1=female) | -1.056 | 0.622 | .091 | [-2.274, 0.165] |
| Boredom dislike | 0.152 | 0.147 | .300 | [-0.136 , 0.440] |
| Boredom frequency | -0.468 | 0.091 | < .001 | [-0.646 , -0.291] |
| Boredom dislike × boredom frequency | -0.262 | 0.080 | .001 | [-0.419, -0.106] |
| **Model with boredom dislike and boredom intensity** | | | | |
| Intercept | 25.940 | 0.439 |  | [25.078, 26.799] |
| Gender (-1=male, 1=female) | -1.079 | 0.624 | .085 | [-2.300, 0.146] |
| Boredom dislike | 0.150 | 0.149 | .315 | [-0.142, 0.441] |
| Boredom intensity | -0.156 | 0.100 | .122 | [-0.352 , 0.041] |
| Boredom dislike × boredom intensity | -0.206 | 0.088 | .019 | [-0.378 , -0.034] |
| **Model with boredom normalcy and boredom frequency** | | | | |
| Intercept | 25.886 | 0.438 |  | [25.025, 26.743] |
| Gender (-1=male, 1=female) | -1.079 | 0.623 | .084 | [-2.299, 0.143] |
| Boredom normalcy | 0.153 | 0.145 | .292 | [-0.132, 0.438] |
| Boredom frequency | -0.441 | 0.090 | < .001 | [-0.618, -0.264] |
| Boredom normalcy × boredom frequency | 0.031 | 0.081 | .700 | [-0.127, 0.189] |
| **Model with boredom normalcy and boredom intensity** | | | | |
| Intercept | 25.900 | 0.438 |  | [25.035, 26.753] |
| Gender (-1=male, 1=female) | -1.085 | 0.623 | .082 | [-2.305, 0.137] |
| Boredom normalcy | 0.100 | 0.146 | .494 | [-0.186, 0.386] |
| Boredom intensity | -0.129 | 0.099 | .193 | [-0.323, 0.065] |
| Boredom normalcy × boredom intensity | -0.007 | 0.093 | .941 | [-0.190, 0.176] |

*Note.* All continuous predictors were within-person centered.

# Testing the Influence of an Item in Boredom Dislike Subscale on Main Results in Studies 1 and 2

The item “Boredom drags down my mood” in the boredom dislike subscale might potentially conflate with mental well-being. To check whether this item disproportionately affects our main results, we conducted the same analyses with a composite of boredom dislike excluding the item “Boredom drags down my mood.” Results stayed largely the same (see Table S9).

**Table S9**

*Regression Models (Study 1) and Random-intercept Models (Study 2) with Mental Well-being as Outcome Variable*

| Predictor | *B* | *SE* | β | *p* |
| --- | --- | --- | --- | --- |
| **Model with boredom dislike and boredom frequency in Study 1** | | | | |
| Intercept | 21.872 | 0.087 |  |  |
| Boredom dislike^a^ | -0.205 | 0.059 | -0.068 | < .001 |
| Boredom frequency | -0.743 | 0.042 | -0.341 | < .001 |
| Boredom dislike^a^ × boredom frequency | -0.077 | 0.025 | -0.057 | .002 |
| *Adjusted R^2^* | 0.132 |  |  |  |
| **Model with boredom dislike and boredom intensity in Study 1** | | | | |
| Intercept | 21.847 | 0.091 |  |  |
| Boredom dislike^a^ | -0.071 | 0.062 | -0.023 | .255 |
| Boredom intensity | -0.741 | 0.046 | -0.333 | < .001 |
| Boredom dislike^a^ × boredom intensity | -0.034 | 0.026 | -0.025 | .194 |
| *Adjusted R^2^* | 0.116 |  |  |  |
| **Model with boredom dislike and boredom frequency in Study 2** | | | | |
| Intercept | 25.408 | 0.313 |  |  |
| Boredom dislike^a^ | 0.176 | 0.141 |  | .214 |
| Boredom frequency | -0.472 | 0.091 |  | < .001 |
| Boredom dislike^a^ × boredom frequency | -0.273 | 0.081 |  | < .001 |
| **Model with boredom dislike and boredom intensity in Study 2** | | | | |
| Intercept | 25.410 | 0.314 |  |  |
| Boredom dislike^a^ | 0.133 | 0.143 |  | .352 |
| Boredom intensity | -0.149 | 0.100 |  | .135 |
| Boredom dislike^a^ × boredom intensity | -0.239 | 0.088 |  | .007 |

*Note.* All predictors in Study 1 were grand-mean centered and all predictors in Study 2 were group-mean centered.

^a^ Composite score of boredom dislike excluding the item “Boredom drags down my mood.”

# Testing a Regression Model with All the Predictors in Study 1

We conducted a regression analysis with well-being as the outcome variable, and boredom dislike, boredom normalcy, boredom frequency, boredom intensity and their interaction terms as the predictors. Results are presented in Table S10.

While these results replicated the findings with separate models, they raised some challenging questions such as what boredom frequency (“How often have you felt bored in the last two weeks?”) and boredom intensity (“When you feel bored, what is your experience of it like?”) mean when their shared variance are partialled out; what the interaction term means when the other interaction term is controlled for. Given the conceptual ambiguity, we retained our analyses that featured separate models in the main text.

**Table S10**

*Regression Model with Mental Well-being as Outcome Variable*

| Predictor | *B* | *SE* | β | *p* |
| --- | --- | --- | --- | --- |
| Intercept | 21.891 | 0.090 |  |  |
| Boredom dislike | -0.128 | 0.065 | -0.041 | .051 |
| Boredom normalcy | 0.303 | 0.070 | 0.081 | < .001 |
| Boredom frequency | -0.512 | 0.050 | -0.235 | < .001 |
| Boredom intensity | -0.426 | 0.054 | -0.192 | < .001 |
| Boredom dislike × boredom frequency | -0.133 | 0.033 | -0.098 | < .001 |
| Boredom dislike × boredom intensity | 0.034 | 0.035 | 0.024 | .328 |
| Boredom normalcy × boredom frequency | -0.005 | 0.041 | -0.003 | .901 |
| Boredom normalcy × boredom intensity | 0.009 | 0.041 | 0.005 | .827 |
| *Adjusted R^2^* | 0.165 |  |  |  |

*Note.* All predictors were centered.

# Testing the Between-person Associations of Boredom Beliefs, Boredom Experience and Mental Well-being in Study 2

We examined the between-person associations of boredom beliefs, boredom experience, and mental well-being with Study 2’s baseline data (*N* = 293). It should, however, be noted that this sample size only afforded a power of .80 to for detecting effects sized *r* = .16, assuming a Type-I error rate of 5% (two-sided), according to sensitivity analysis. Based on the effect size of the interaction (β = -0.081) we found in Study 1, a minimum sample size of 1,199 participants, with power of .80, is needed to detect this effect with an alpha of .05.

To test Hypothesis 1, we examined the zero-order correlations between boredom dislike, boredom frequency and boredom intensity. For Hypothesis 2, we conducted regression analyses to examine whether mental well-being was predicted by boredom dislike (or boredom normalcy), boredom frequency (or boredom intensity), and their interaction terms.

Supporting Hypothesis 1, boredom dislike was positively associated with frequency (*r* = .19, *p* = .001; H1a) and intensity (*r* = .20, *p* < .001; H1b) of boredom. For Hypothesis 2, results of the regression analyses are presented in Table S11. Mental well-being was significantly associated with boredom frequency (or boredom intensity) but not with boredom dislike and their interaction term (H2a & H2b). These results are different from what we found in Study 1. This might be attributed to the differences in sample sizes (Study 1’s *N* = 2,495, Study 2’s *N* = 293), and thus reduced power in detecting the interaction. Moreover, regression analyses with mental well-being as the outcome variable revealed a negative main effect of boredom frequency (or boredom intensity) and a positive main effect of boredom normalcy; there was no significant interaction between boredom frequency (or boredom intensity) and boredom normalcy. These results replicate the findings from Study 1.

**Table S11**

*Regression Models with Mental Well-being as Outcome Variable in Study 2’s Baseline Data*

| Predictor | *B* | *SE* | β | *p* |
| --- | --- | --- | --- | --- |
| **Model with boredom dislike and boredom frequency** | | | | |
| Intercept | 24.790 | 0.295 |  |  |
| Boredom dislike | -0.101 | 0.181 | -0.033 | .577 |
| Boredom frequency | -0.562 | 0.128 | -0.253 | < .001 |
| Boredom dislike × boredom frequency | -0.064 | 0.069 | -0.053 | .355 |
| *Adjusted R^2^* | 0.059 |  |  |  |
| **Model with boredom dislike and boredom intensity** | | | | |
| Intercept | 24.745 | 0.301 |  |  |
| Boredom dislike | -0.122 | 0.183 | -0.039 | .507 |
| Boredom intensity | -0.413 | 0.147 | -0.166 | .005 |
| Boredom dislike × boredom intensity | -0.002 | 0.075 | -0.001 | .982 |
| *Adjusted R^2^* | 0.022 |  |  |  |
| **Model with boredom normalcy and boredom frequency** | | | | |
| Intercept | 24.742 | 0.284 |  |  |
| Boredom normalcy | 0.723 | 0.187 | 0.214 | < .001 |
| Boredom frequency | -0.576 | 0.123 | -0.260 | < .001 |
| Boredom normalcy × boredom frequency | 0.034 | 0.078 | 0.024 | .660 |
| *Adjusted R^2^* | 0.103 |  |  |  |
| **Model with boredom normalcy and boredom intensity** | | | | |
| Intercept | 24.749 | 0.290 |  |  |
| Boredom normalcy | 0.681 | 0.191 | 0.202 | < .001 |
| Boredom intensity | -0.400 | 0.143 | -0.161 | .006 |
| Boredom normalcy × boredom intensity | 0.049 | 0.090 | 0.032 | .585 |
| *Adjusted R^2^* | 0.063 |  |  |  |

*Note.* All predictors were centered.

# References

Cheung, G. W., & Rensvold, R. B. (2002). Evaluating Goodness-of-Fit Indexes for Testing Measurement Invariance. *Structural Equation Modeling: A Multidisciplinary Journal*, *9*(2), 233–255. https://doi.org/10.1207/S15328007SEM0902_5

Hu, L., & Bentler, P. M. (1999). Cutoff criteria for fit indexes in covariance structure analysis: Conventional criteria versus new alternatives. *Structural Equation Modeling: A Multidisciplinary Journal*, *6*(1), 1–55. https://doi.org/10.1080/10705519909540118

Putnick, D. L., & Bornstein, M. H. (2016). Measurement invariance conventions and reporting: The state of the art and future directions for psychological research. *Developmental Review*, *41*, 71–90. https://doi.org/10.1016/j.dr.2016.06.004
